# “Feature Detection” vs. “Predictive Coding” Models of Plant Behavior

**DOI:** 10.3389/fpsyg.2016.01505

**Published:** 2016-10-04

**Authors:** Paco Calvo, František Baluška, Andrew Sims

**Affiliations:** ^1^Minimal Intelligence Lab (MINT Lab), Department of Philosophy, University of MurciaMurcia, Spain; ^2^School of Philosophy, Psychology and Language Sciences, School of Biological Sciences, University of EdinburghEdinburgh, UK; ^3^Institute of Cellular and Molecular Botany, University of BonnBonn, Germany; ^4^Institut Supérieur de Philosophie, Université Catholique de LouvainLouvain, Belgium

**Keywords:** plant behavior, predictive coding, feature detection, plant perception, root transition zone

## Abstract

In this article we consider the possibility that plants exhibit anticipatory behavior, a mark of intelligence. If plants are able to anticipate and respond accordingly to varying states of their surroundings, as opposed to merely responding online to environmental contingencies, then such capacity may be in principle testable, and subject to empirical scrutiny. Our main thesis is that adaptive behavior can only take place by way of a mechanism that predicts the environmental sources of sensory stimulation. We propose to test for anticipation in plants experimentally by contrasting two empirical hypotheses: “feature detection” and “predictive coding.” We spell out what these contrasting hypotheses consist of by way of illustration from the animal literature, and consider how to transfer the rationale involved to the plant literature.

## Introduction

Sculpted by evolution, plants allegedly react to environmental inputs only in an instinctual manner, with their behavioral repertoire reducing to invariant tropistic (directional) or nastic (non-directional) responses implemented in the form of sets of fixed reflexes (Silvertown and Gordon, 1989; Trewavas, 2009, 2014). This behavior is usually accounted for in hard-wired terms, and being hard-wired undermines the ascription of intelligence to plants. That is because behavioral flexibility is one marker of cognitive sophistication, and hard-wired behavior does not admit of behavioral flexibility; it is “hard-wired” to particular stimuli or cues. This mechanistic and non-cognitive view of plant behavior goes back to Julius von Sachs and Jacques Loeb (Greenspan and Baars, 2005)—incidentally, Loeb introduced this concept to animal behavior from his earlier studies on plants, proposing similar hard-wired explanations of both animal and plant behavior (Loeb, 1918).

This reductionist approach is likely to fail in animal biology (Greenspan and Baars, 2005; Greenspan, 2012) and, we contend, is equally likely to fail in plant biology (Trewavas, 2005, 2009, 2014; Karban, 2008; Baluška and Mancuso, 2009a,b, 2013; Calvo Garzón and Keijzer, 2011; Trewavas and Baluška, 2011; Marder, 2012, 2013; Gagliano et al., 2014; Cvrčková et al., 2016). For one thing, plants are motile, and their behavioral repertoire is richer than commonly acknowledged. Virtually no growing part of any single plant fails to exhibit a movement of nutation (Mugnai et al., 2007). Shoots of climbing plants guide their movements to reach a support; roots navigate belowground, guiding their movements to secure nutrients intake; young and terminal leaves display helical and rotational oscillatory movements, etc. (Darwin, 1875; Darwin and Darwin, 1880). In fact, sophisticated forms of plant behavior abound. Plants and their roots are sensitive to a variety of signals other than water, light, minerals, or gravity. Plants can sample more than 20 different biotic and abiotic parameters from their environment and integrate this complex sensory information to mount appropriate behavioral responses (Knight et al., 1998; Karban, 2008; Hodge, 2009; Trewavas, 2009; Baluška, 2012; Baluška and Mancuso, 2013). Roots grow by assessing the future acquisition of minerals and water, a process that requires the integration of gravity, moisture and mechanical perturbations, among other vectors (Takahashi et al., 2002). Likewise, roots can, for instance, sense available space and discriminate self-roots from alien roots. Less familiar examples include salt-avoidance behavior (Li and Zhang, 2008; Sun et al., 2008; Yokawa et al., 2014). Here salinity interacts with the gravitropic response and an overall integrated signal assessment appears to be needed in order to optimize growth under abnormal saline conditions.

Unveiling why plant behavior is so flexible (Trewavas, 2009, 2014) may cast a new light on intelligence without recourse to anthropo- or zoomorphisms. In this context, our theoretical motivation in this article is to consider the possibility that plants exhibit anticipatory behavior, a mark of intelligence. If plants are able to anticipate and respond accordingly to varying states of their surroundings in a context-sensitive way, as opposed to merely responding online to environmental contingencies, then that capacity may be in principle testable, and subject to empirical scrutiny. Our main thesis is that adaptive behavior can only take place by way of a mechanism that *predicts* the organism's own states of sensory stimulation (Egner et al., 2010; Chennu et al., 2013).

More broadly, we regard (minimal) cognition to be a biological phenomenon. What seems to be involved is having a sensorimotor organization, and free-moving with the purpose of manipulating the environment (allowing for metabolic forms of adaptation and anticipatory functioning) (Calvo Garzón and Keijzer, 2011). This holds for all forms of life. It is the ability to act upon environmental contingencies that defines biological systems. Only those biological systems can survive which perceive the world veridically via successfully predicting it, and not merely reacting to it (Clark, 2016). Of course, the falsification of a reactive model would not imply that a particular anticipatory countermodel is correct. The notion of anticipation may come in a variety of forms, with weaker and stronger readings being possible. Anticipatory behavior may rely upon the capacity of the system to model internally the environmental sources themselves. But forms of anticipation according to which predictive success is a function of actual past behavior—stronger forms of anticipation that do not depend on modeling the future internally (Stepp and Turvey, 2010; Stepp et al., 2011)—cannot be discarded beforehand^1^. However, for present purposes we are proposing a test for anticipation in plants experimentally by contrasting two empirical hypotheses exclusively: “feature detection” and “predictive coding (processing).”

According to the feature detection hypothesis, plants perceive their surroundings, but the detection of and adaptive responses to environmental features are consistent with a reactive interpretation of plant behavior. By contrast, the capacity to perform predictive processing would point toward a basic form of agency: plant perception may be seen as an active process of probabilistic inference akin to that found in animals (Kok et al., 2013). Plants under this interpretation are pro-active; they actively sample their environment to generate information, estimating the likelihood that one external state of affairs, and not another, is the source of energy impinging upon its sensory periphery.

Now, there is a preliminary question that might be raised as to whether anticipatory behavior entails intelligence. Our position is not that such behavior is *sufficient* for intelligence; the existence of basic predictive abilities in artificial neural networks would seem to rule that out. But we do contend that such behavior is *necessary* for the existence of minimal intelligence, which is to say that it is a general feature of it. Therefore, our proposed study contributes to the question of minimal intelligence in eukaryotes in the following way: if the feature detection model of plant behavior is confirmed by the study, then we can claim to have falsified the minimal intelligence hypothesis. But if it is not then we can provisionally retain the conjecture.

In the following sections we spell out what these contrasting hypotheses consist of by way of illustration from the animal literature, and consider how to transfer the rationale involved to the plant literature. Discussion and directions for future research will follow.

## Expectation and surprise in the ventral visual pathway

The canonical domain for feature detection is visual cognition. The origin of the feature detection hypothesis dates back to Barlow's (1953) work on the frog's retina, according to which single ganglion cells could release particular motor acts triggering specific behaviors. Barlow's insight was elaborated further by Lettvin et al. (1959), who showed how different ganglion cells responded to different patterns of excitation, and subsequently by Hubel and Wiesel (1965) who extended the paradigm to mammalian research^2^. According to the feature detection model, neurons are seen as specialized bottom-up feature detectors that respond selectively to, for example, angles, lines, movement, or edges. Information flows upwards into deeper visual cortical layers (V2, V4, IT), allowing for the receptive field of neurons within those layers to respond to stimuli of increasing complexity.

By contrast, information in the “predictive coding” model (Rao and Ballard, 1999; Friston, 2005; Egner et al., 2010; Chennu et al., 2013) flows simultaneously in both directions as well as laterally within individual levels of processing. The nature of these two “flows” is as follows. Firstly, predictions (conditional probabilities of particular features being the cause of stimulation) are propagated top-down. Secondly, mismatches between those predictions and the incoming input signals (prediction errors) are propagated bottom-up. At each level in the hierarchy, predicted inputs are compared with actual inputs and the latter are only propagated upwards if there is a mismatch, i.e., a prediction error. The aim is to minimize prediction error (the difference between the top-down prediction of what the sensory signal is and the actual signal traveling bottom-up). In this way, perception is interpreted as the end result of a process whereby top-down predictions match the environmental input, based on a hierarchical generative model of the *causes* of that input.

It is possible to test between “feature detection” and “predictive coding” in animal visual cognition. The two models generate different predictions and thus are subject to empirical contrast. In particular, Egner et al. (2010) designed an experiment to check for interaction between expectations and error (degree of surprise), as opposed to simple feature detection. In their study, they considered the fusiform face area (FFA), a visual area located in the ventral system known to be involved in facial recognition. Were “feature detection” to be correct, the FFA area would respond to stimulus facial features *per se*. On the other hand, if “predictive coding” is correct, the FFA area should respond to the weighted sum of expectation and surprise: the sum of top-down predictions (say, the expectation to see a face) and bottom-up surprise (the degree of expectation violation as a face is fed to the system, or not).

**Figure 1:**
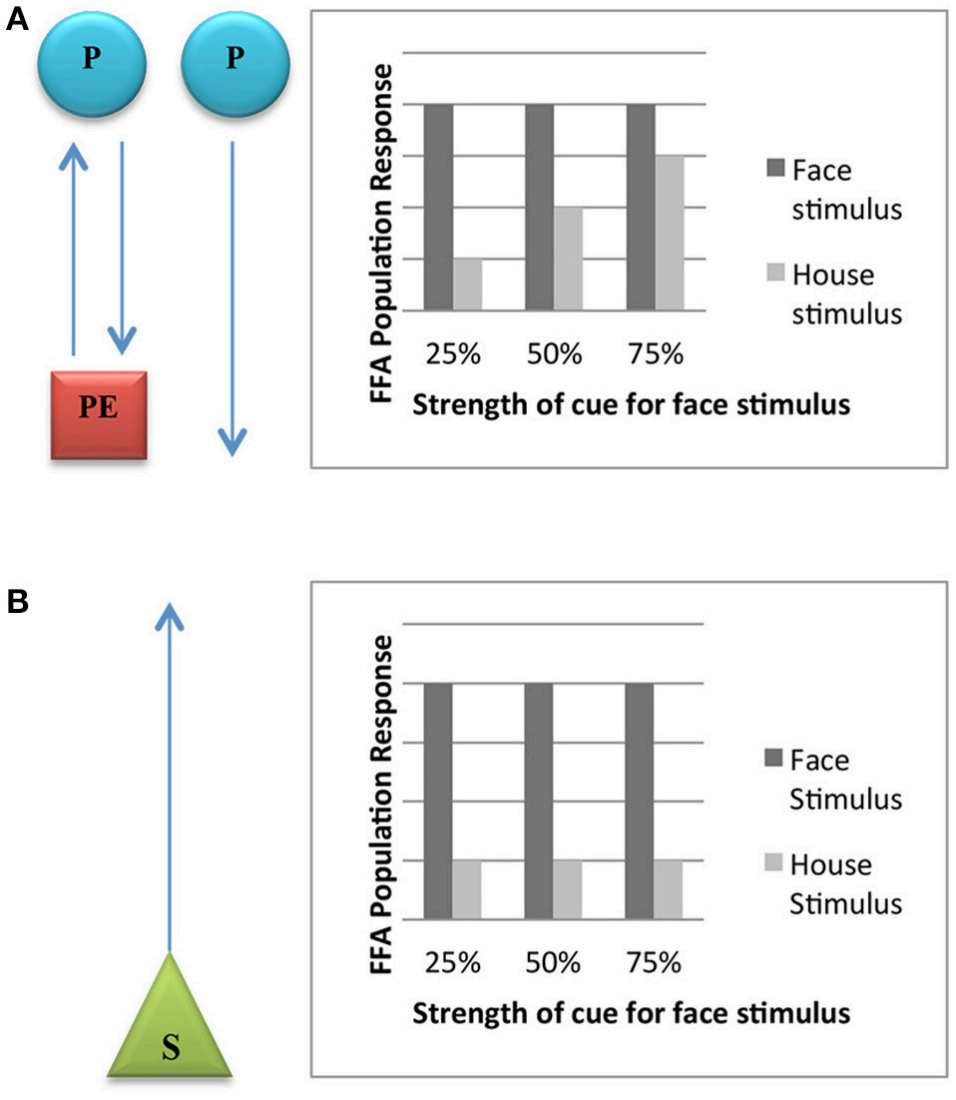
**(A) Expected FFA population response on the predictive processing hypothesis**. The diagram on the left shows that the summed FFA population response should reflect both prediction (P) and prediction error (PE), unless there is no prediction error present due to the prediction being correct. The graph on the right demonstrates the prediction of FFA response if the hypothesis is true. Because there the cues elicit the expectation in the subject that a face will be displayed, the FFA is active in trials for which the house is displayed. **(B)** Expected FFA population response on the feature-detection hypothesis. The diagram on the left shows that the summed FFA population response should only reflect actual stimuli (S). The graph on the right demonstrates the prediction of FFA response if the hypothesis is true.

Egner et al. (2010) tested these hypotheses with fMRI measurements by varying both the stimulus features (pictures of faces vs. pictures of houses) and the expectations generated by experimental subjects as the material is being presented to them. They did so by pairing the stimulus features (faces and houses) with colored frames (green, yellow, blue), in such a way that the frames provided the low, medium or high cue (25, 50, 75%) that the incoming stimulus would be a face). The prediction entailed by the predictive processing model is that whereas faces and non-faces will elicit equivalent FFA responses when subjects have a high face expectation (since in both cases there is no association with activity related to face surprise), faces and non-faces will elicit maximally differing FFA responses when subjects have a low face expectation (only one condition, faces, is associated with face surprise)^3^. By contrast, the prediction entailed by the feature detection model would be different FFA responses under both conditions since subjects are exposed in a strict bottom-up fashion to face features or non-face features, regardless of the expectations involved. These results are plotted, respectively, in Figures 1A,B.

Would plants also be able to generate expectancies in line with the interpretation that obtains in Figure 1A? In the following sections we argue that this is an open empirical possibility.

There is an additional motivation for applying a seemingly zoo-centric theory to the understanding of plant behavior. That is the nature of predictive processing in cognitive neuroscience as a specific application of a more general theory of *biological* functioning. This more general theory is called the *free energy principle* (Friston, 2010, 2013; Hohwy, 2015; Seth, 2015). The free energy principle applies to all biological self-organizing systems, and it is a hypothesis about how it is that such systems manage to keep within the state-space that constitutes homeostasis for that system. On the free energy principle, it is the ability to act upon its environment to avoid damaging phase changes—to keep within this state-space—which defines specifically biological systems.

An example used to illustrate this idea is given by Friston and Stephan (2007, p. 423). Their example of a living/non-living pair is that of a bird and a snowflake. Each of these systems is comprised by a physical structure which entails a set of states within which that system must remain in order to maintain homeostasis. The snowflake, for example, will melt if it encounters temperatures above a certain point. The same applies to the bird phenotype, though the complexity and resilience of that phenotype implies a much wider range of possible states which it can occupy. The difference between the bird and the snowflake is that the bird interacts with its local environment in order to stay within its optimal state-space: it seeks nutrition, avoids predators, and moves around within its ecological niche.

Another way of putting this difference is that the biological agent must avoid states that are *surprising*. These states are not surprising in the phenomenological sense of the word, but are rather improbable given some implicit set of expectations. That is to say that they are surprising in an information-theoretic sense. In our example above, the implicit expectations in question are constituted by the phenotype of the organism. Surprising states for a snowflake are those in which it melts; surprising states for a fish include those in which it is too dry to survive (cf., Hohwy, 2015, pp. 3–4).

The free energy principle states that biological agents can perform this task by way of free energy minimization, which is an upper bound on surprise; more specifically, that “biological systems on average and over time act to minimize free energy” (Hohwy, 2015, p. 2). That is a process that is mathematically analogous to prediction error minimization as it is construed in predictive processing (free energy is equal to the long-term sum of prediction error). That means that the free energy principle implies that the organism maintains a generative model (it generates predictions of future states) of the causes of its sensory states, and works at minimizing prediction error with respect to this model.

There are two ways in which prediction error minimization can proceed. The first of these could be called perceptual inference. In perceptual inference, prediction error is minimized by updating expectations in order to bring them into line with actual states, rather than those that were falsely predicted by the expectations before updating. This is the process that is thought to explain performance in the visual perception tasks discussed above (Egner et al., 2010). The other possible way of minimizing prediction errors is known as active inference. In active inference, prediction error is minimized by the selective sampling of sensory states such that those states are brought into line with what is predicted by prior expectations. It is this process that is thought to explain the function of visual saccading, in which the eye moves rapidly and to take in different parts of the visual field. The active-inferential theory of visual saccading states that these saccades constitute the sampling of sensory states in such a way that evidence is gathered for predictions about visual input (Friston et al., 2012a)^4^.

We can cash out this quasi-cognitive distinction between perceptual and active inference in a biological context by referring to the example of a fresh-water fish. These fish cannot survive in salt water. That means that the cascade of sensory input that immersion in salt water elicits is highly surprising to that biological agent. Such immersion will generate a great deal of prediction error, or free energy. In order to minimize that free energy the fish can do one of two things. The first is to re-sample the environment such that future input is brought back into line with expectations. That means active inference; it means that the fish follows the salt-water gradient back into fresh water. The second is to update its expectations such that it expects to be in salt-water. This is perceptual inference; that likely happens initially and at some level in the predictive hierarchy; though given the relatively unlikely state that immersion in salt water represents, it is not to be ultimately chosen as an adaptive strategy.

This entails a link between homeostasis and anticipative behavior. That is to say, the free energy principle entails the hypothesis that the mechanism by which biological systems maintain homeostasis is a kind of anticipation; namely, by minimizing the difference over time between the implicit model of the world that its morphology constitutes and the sensory states that it finds itself in. That means the minimization of surprise, and this is done through perceptual inference (morphological change in response to sensory states, ranging from different patterns of activation across the visual cortices in mammals all the way down to hardening responses to abiotic stresses in plants) as well as active inference (selective sampling of sensory states through movement in the environment, such as visual saccading in animals and nutations in plants). For this reason the predictive processing theory is a prime candidate for the explanation of plant behaviors of the kind we label “minimally intelligent” in this article. The analogous and wider-scoped free energy principle provides one rationale for spreading wider the scope of explanatory concepts that have heretofore been confined to application in animal biology.

Lastly, there is an important distinction in the predictive coding literature between two kinds of learning architecture: model-based and model-free (Daw et al., 2005). Model-based architecture follows predictive coding as it has already been described; prediction error is minimized relative to a hierarchical generative model of the causes of sensory states. So it is representation-heavy mode of predictive coding that implies a relatively sophisticated model of the environment; the responses of the organism to the environment are mediated through the model. Model-free architecture lacks any such model; the responses of the organism to the environment are direct and unmediated. A model-free architecture will produce learning responses that proceed through trial and error, like classical reinforcement learning. Whether plant learning is model-based or model-free (or both) would have interesting consequences for our hypothesis. If plant learning includes model-based strategies, then it is much more sophisticated than formerly assumed, and capable of making deeper and more discriminative distinctions between different kinds of stimuli. We may therefore further massage our hypothesis into two distinct commitments: a weaker, on which plant cognition is entirely model-free; and a stronger, on which it also includes model-based learning. We consider how we might empirically distinguish between these later on in the paper.

By way of a *caveat*, it must nonetheless be noted that not all the details of the free energy principle are worked out here, and it is indeed not uncontroversial as a whole (e.g., Gershmann and Daw, 2012). On the other hand, and before we consider further the predictive processing/free-energetic vs. the feature-detection interpretation of plant behavior, it must be borne in mind that despite features themselves being hard-wired, there is room for learning under the feature detection paradigm. In effect, the release of specific motor acts/behavioral patterns via, for example, single retinal ganglion cells in Barlow's (1953) original model is consistent with the possibility that learning takes place: the events being coded for by detectors could well be hard-wired, and yet the mapping between the features that are being detected and subsequent actions be learned^5^. As a result, we would be confronted with two different models of how a system successfully anticipates environmental contingencies, both sculpted via learning. For present purposes, we shall thus restrict our attention to the Barlow (1953), Lettvin et al. (1959) and Hubel and Wiesel (1965) original learning-free formulation, for the sake of contrasting it with predictive processing; a model that connects anticipation with learning courtesy of an internal model subsequently exploited for the purpose of generating predictions. As to the free energy principle, it is sufficiently plausible, and its closeness to predictive processing gives us cause to think that the empirical tests that we describe are worth carrying out in the context of plant movements. Indeed, those prospective studies could be construed as a contribution to the wider project of putting the free energy principle to the test in an experimental setting.

## Applying the experimental paradigm to plants

If the predictive processing/free-energetic interpretation of plant behavior is on the right track (Figure 1A), then we should be able to observe plant excitable cells responding to the summation of activity related to expectation. Or, at the plant organ/root level, the actual context of the environmental situation should determine the behavioral outcome as roots explore their surroundings. By contrast, should feature detection be correct, then we should expect no interaction with expectations or context. In principle, we should observe plant cells and organs responding exclusively to the physical vector of stimulation.

In order to make these two hypotheses testable, a number of options are available with regard to (i) the type of gradient potentially being sensed (gravity, light, moisture, oxygen, touch, etc.), and (ii) the experimental method by which to generate the required expectations. Finally, (iii) different measurement techniques may be considered when testing our working hypotheses.

(i) As to the type of stimuli, we may consider either the perception of a single modality (for example, gravisensing) or the integrated response to several vectors, such as the weakening or inhibition of the response to gravity when stimulated by touch (Fasano et al., 2002), light (Liscum, 2002; Wan et al., 2012), water (Takahashi et al., 2002), or sound waves (Gagliano et al., 2012). As well, such stimulations may be continuous or transient. Continuous touch stimulation may obtain by placing a barrier that prevents root gravitropism. Or we may consider continuous bioacoustic stimulation (Gagliano et al., 2012). By contrast, in the case of transient touch stimulation, we may consider, for instance, the stimulation of root cap peripheral cells (Massa and Gilroy, 2003), or transient bioacoustic stimulation.

(ii) With respect to the manipulation of the degree of expectation, there are different options. In the aforementioned case of visual cognition and FFA, they achieved it by pairing the stimulus features (faces and houses) with colored frames (green, yellow, blue) in such a way that the frames provided the cue (low, medium or high) that the incoming stimulus would be a face (Egner et al., 2010). Likewise, both stimulus features for the experimental setting in plants, and the expectation of encountering those stimulus features, would need to be varied independently.

In order to generate expectations we can either use the same or a different network. That is, the cues that precede the presentation of the stimuli can belong to the same type of system or to a different one, such as happens in the integration of electrical network with chemical network systems when electrical activity is elicited by chemical means. Expectations may also be generated by mechanical stimuli, elicitation of electric spikes, firing with transient stimulation, and repetition (e.g., transient acoustic stimulation).

(iii) Finally, with regard to the available measurement techniques, we may use the measurement of behavioral events (for instance, root bending, and tropism), single-cell recordings, or non-invasive neuroimaging techniques.

To take a particular example for the sake of illustration, consider the phenomenon of “repetition suppression,” where it is found that the repeated presentation of a stimulus results in the attenuation of neural activity (Todorovic et al., 2011). In an MEG study, Todorovic et al. (2011) presented human subjects with auditory stimuli whose repetition were manipulated so that they were subject to different levels of expectation. They reported that a larger suppression is registered when subjects expect the repetition to take place. This is observed in auditory cortex activity and synchrony. In their view, repetition suppression is to be interpreted in predictive coding terms: top-down expectations underlie suppression. What that means is that as the stimulus in question comes to be expected, it becomes part of the model that is being used to predict the flow of sensory information. This means that presentation of that stimulus generates less prediction error, less stress penalty, and therefore less activity, since it is already integrated into the top-down signal which will not be contradicted by mismatch.

Considering that repetition suppression is a robust phenomenon that has been observed at different time scales in a number of different sensory modalities, and in response to a variety of stimulus properties (Grill-Spector et al., 2006), it is a possibility that the phenomenon also exists in plants. In support of this scenario, osmo-sensory potentiation has been reported recently. Stephan et al. (2016) have found rapid onset and reversibility of sensory potentiation via plastid-mediated calcium spikes primarily in root apices.

In line with Todorovic et al. (2011), we could test for the possibility that the response in terms of activity and synchrony of transition zone (TZ) cells is attenuated under conditions of expected stimulus repetition (TZ is a root apex region interpolated between the apical meristem and sub-apical cell elongation region that is specialized in processing and integrating sensory information, and instructing the motoric regions driving goal-directed avoidance and exploratory root tropisms—see Baluška et al., 2009, 2010; Baluška and Mancuso, 2013). If repetition is unexpected, by contrast, then we should expect that TZ cell responses would undergo less attenuation. The predictive coding explanation would be that there is less prediction error under expected experimental conditions. Likewise, unexpected omissions may result in a rise in synchronous activity (Baluška and Mancuso, 2013) over TZ cells.

If we were to draw an analogy between transition zone cell response in a study like this and the activity of the FFA in Egner et al. (2010), then we might be construing transition zone cell response as correlated with the levels of prediction error that are generated in response to the presented stimulus—i.e., the mismatch between expected states and sensory states. Alternatively, we might be construing the discrepancy in attenuation as a function both of prediction and prediction error (see Egner et al., 2010). But in either case, we need the mismatch between expectation and sensory states in order to give the explanation.

Behaviorally relevant measurements in bioacoustics may involve the angle of root bending induced by auditory cues that have different probabilistic relationships with the distribution of the sources of stimulation (low frequencies of 200 Hz corresponding to water stream, as opposed to higher frequencies—Gagliano et al., 2012). Or consider water and nutrient availability at the root level. One could use a simple binary maze system, allowing maize roots to take decisions in their growth direction that are stimulated or inhibited by well-defined chemicals, plant growth regulators, and plant nutrients (Yokawa et al., 2014). Growing maize roots make robust decisions on the basis of the available chemicals, and an experimental set-up would allow further analysis of root decisions and learning/memorization phenomena in plants. It is technically possible to increase the number of possible choices by increasing the number of available paths containing stimulants or repellents for root growth. Besides chemical stimuli, one could also load some paths using physical stimuli such as light or sound (mechanical vibrations). In our previous study, we reported that maize roots grow vigorously inside of glass capillaries even when these are inversed and root grow up against the gravity vector (Burbach et al., 2012). However, as soon as they are exposed to light, roots accomplish promptly U-turn movements via complicated thinning of their apices (maize roots are too thick for turning in these thin capilaries), and grow down the gravity vector (Burbach et al., 2012). Similarly, maize roots growing on hard substrate via their characteristic “crawling” movements in darkness stop this and try to grow down the gravity vector if exposed to light. This photophobic root behavior, similar to the root “crawling” movements, requires intact root caps. Decapped roots grow vigorously but are unable to behave (Burbach et al., 2012). Since light acts as stressor to plant roots, we proposed that this is not just a negative phototropism but rather an active escape tropisms (Yokawa et al., 2011, 2013; Burbach et al., 2012; Xu et al., 2013). This fast root response to sudden illumination implies that maize roots are well prepared to this stress situation and respond very fast to escape back into the darkness. Similarly, placing roots horizontally induces very rapid (within a few seconds) gravistimulated root bending, again implying that roots are well prepared for such a situation (Baluška and Volkmann, 2011).

The next well-studied phenomenon, related to memory-based plant behavior, is stress memory in plants (Bruce et al., 2007). As discussed below, repetitive exposure of plants to particular stress situations is well known to be memorized by plants as these “expect” the next stress challenge and are already prepared to cope better with these stress challenges (Knight et al., 1998; Goh et al., 2003; Bruce et al., 2007; Harb et al., 2010).

Particularly instructive might be measurements of synchronous oscillations at the root apex transition TZ zone (Baluška and Mancuso, 2013). The idea is that synchrony patterns determine impacts of the top–down feedback flows. Such analysis will allow us to test whether plant nutrients and relevant stimuli differ from non-nutrients and non-relevant stimuli in their elicitation of similar TZ cell responses when roots have a high nutrient (or specific stimuli) expectations. The idea is to adjudicate between our working hypotheses by registering data from transition zone action potentials while independently varying both stimulus features (e.g., 200 Hz vs. 1000 Hz) and plant expectations regarding those features (low, medium vs. high water 200 Hz expectation). If the responses elicited are similar, then that would serve as support for the predictive coding hypothesis. In contrast, should different transition zone responses be obtained, this would support the feature detection hypothesis (based on the strict bottom-up responses to nutrient features or non-nutrient features).

Taking into account the distinction between model-based and model-free modes of predictive coding as it was defined at the end of Section Expectation and Surprise in the Ventral Visual Pathway, we should note at this point that the empirical tests that we suggest here would not distinguish sufficiently between these. That is because the “anticipation” that is assayed is of a kind analogous to the Egner et al. (2010) study. The paradigm of Egner and his collaborators is not sensitive to a distinction between model-free and model-based learning, since either of these could produce the relevant sensitivity to context (though given antecedent commitments about human vision, a model is assumed). How, then, would experimenters working with plant models be able to claim support for the weak (entirely model-free) or strong (model-based, or mixed) hypothesis?

Daw et al. (2011) have designed a paradigm for human subjects which can distinguish between model-based and model-free learning processes. The experimental participant performs a sequential two-alternative forced-choice task that has two stages. What is presented in the second stage depends on the participant's choice in the first: each option has a 70% chance of leading to one of two alternative choices in the second stage. The choice in the second task may or may not yield varying levels of reward. Model-based and model-free learning processes will assign value differently in this task, allowing one to distinguish between them. A model-free process will infer a direct linear relation between any reward from the second choice and whatever option in the first choice led to the second, while a model-based learning process will also learn the frequencies with which the first task leads to one or the other second-stage choice, and this will bear on how value is assigned in the case of reward. So if the choice made in the first task leads to that of the two subsequent choices which is less likely given the first choice, and the second choice yields reward, then a model-based learning process will assign value to the first option that was *not* chosen (on the basis that this is more likely to lead to the choice that has just been rewarded), while that model-free process will assign value to the first option that was actually chosen. This is because the model-based process is able to recognize the statistical relations between the first and second stages.

Although this paradigm is highly human-centric, we see no principled reason why the basic abstract principle at work in it could not be applied in the case of plant-based paradigms—especially seeing as that the Egner paradigm is just as human-centric. The abstract point is that model-based learning processes can learn probabilistic relations between items in a sequence while model-free learning cannot. We believe that an analogous model could be implemented in plant models. This requires (i) two statistically related items; (ii) a reward associated with the second item; and (iii) an appropriate measuring technique for the assignment of value. This would be a fruitful area for future research.

## Discussion and outstanding questions

Can plants be astonished by the unexpected? Our working hypothesis, backed by recent studies, is that plants have the capacity to make good predictions regarding forthcoming sources of stimulation, whose perception involves the continuous match of expectations against the impinging environmental signals. We propose here to empirically test plant intelligence by contrasting two alternative hypotheses. According to the feature detection hypothesis, plants are passive, hard-wired, and stimulus-driven organisms. They simply react to environmental stimuli in an online, bottom-up fashion. In that case, there seems to be little need to call such form of reactive behavior “intelligent.” According to the second hypothesis, predictive coding, plants rely upon their own expectations in a top-down fashion, and the system stabilizes only once those expectations face the incoming signals which provide the system with a measure of error.

What are the implications for our understanding of the way plants perceive the world? If the predictive coding hypothesis finds empirical support, plant perception is an active process, where the organism/plant constructs its own plant-specific world-view (courtesy of previous ontogenetic exposures coupled with phylogenetic endowment) via the sensory vectors impinging upon the system. Whenever such predictive sensory vectors (the most likely sources of perturbation in a particular context: nutrients, water, etc.) match the incoming environmental signals (the fidelity of generated world-view is high), we may say that active plant *perception* takes place.

Plant root navigation is based on sensory signal integration allowing roots to search for water and nutrients, as well as avoiding/escaping dangerous root patches, in complex and heterogeneous soil environment. As we have discussed previously, the root apex TZ is specialized for such sensory signal integration and also allows effective sensorimotor coupling as the basal cells of this zone control root bendings during root tropisms (Baluška et al., 2009; Baluška and Mancuso, 2013). As cells in this special root apex zone are unique with respect of their high demand of oxygen, which is linked to the endocytic vesicle recycling sensitive to brefeldin A (Baluška and Mancuso, 2013), one can expect that this zone represents some sort of command center responsible for sensorimotor circuits akin to the Darwinian brain of lower animals (Baluška et al., 2009).

Now, if we were able to tell apart our two working hypotheses experimentally, then we envisage that a number of outstanding questions would be forthcoming:

First, the experiment from the animal literature reported here suggests that neurons respond to a mixture of expectation and surprise, which is consistent with predictive processing insofar as *bidirectionality* and *functional asymmetry* (top-down expectations matching bottom-up signals) are needed (Friston, 2005). If the predictive processing hypothesis is correct, then the plant anticipatory system it should be reflected in some form of functional asymmetry. Such an asymmetric functional composition, however, need not imply any anatomically marked difference in between types of cells (for instance, deeper excitable cells sending out their expectation values, with error signals being forwarded in turn). When we talk about deeper and less deep populations, we have in mind cortical layers. But the integration of top-down and bottom-up informational flows need not reside in anatomically marked distinctions as in the hierarchically layered structure of the mammalian cortex. The hypothesis is that anticipation might be embodied in the temporal structure of the synchronous oscillatory activity of the TZ cells. But first we need to characterize TZ oscillations under control and sensory-loaded situations. Next, one can apply repeated trains of well-defined coupled stimuli, e.g., cold stress following particular light stimulation, or cold stress not preceded with such “light warning.” In animals, Engel et al. (2001) argue that cognition is explanatorily grounded in the dynamic bounding of cell populations from the same and from different neural systems. Sensorimotor coordination requires the soft and functional assembling of many different subsystems, and this is achieved courtesy of the synchronous oscillatory nature of populations of neurons (for another option, see Damasio's “convergence-zone” model; Damasio, 1990). In plants, top-down influences can be couched in dynamical terms. In the same way that “large-scale dynamics can have a predominant influence on local neuronal behavior by “enslaving” local processing elements” (Engel et al., 2001), we may interpret synced populations of neurons as entraining those out of sync in order to assemble together and enhance their firing saliency. The capacity of plants to anticipate stress situations might be implemented in the temporal structuring of transition zone activity patterns. Sensorimotor integration might therefore be accounted for by transition zone oscillations (Baluška and Mancuso, 2013).

Second, predictive processing in the mammalian brain is thought to take place hierarchically, as top-down and bottom-up sources of information interact at different spatiotemporal scales throughout the network. For the system to successfully anticipate environmental contingencies, an internal model is sculpted via *learning*; a model that is subsequently exploited for the purpose of generating predictions. What forms of learning might then play a functionally equivalent role in plants? Here, forms of learning such as habituation (Gagliano et al., 2014) look promising. Plants are well known to “learn” from previous stress challenges via modifying their metabolism, cell structures, anatomy and physiology known in plant sciences as hardening or acclimation (Knight et al., 1998; Goh et al., 2003; Bruce et al., 2007; Harb et al., 2010; Dietz, 2014; Ding et al., 2014; Kleinmanns and Schubert, 2014; Shi et al., 2015). Well accepted mechanisms for these plant memory phenomena are linked to epigenetics which is based on heritable chromatin modifications via histone markings (Ha, 2013; Iwasaki and Paszkowski, 2014; Kleinmanns and Schubert, 2014). Similar memory models are well characterized also for animals (Campos et al., 2014). Importantly, epigenetics is relevant not just for cellular levels, but also controls behavior in animals and humans (Impey, 2007; Graff and Mansuy, 2008; Peixoto and Abel, 2013). Besides epigenetic-based memories, however, plants might also use synaptic-like supracellular memories. This is suggested by the fact of immunological memories, which have already been reported to exist in plants since 1986 (Baldwin and Schmelz, 1986; Ruuhola et al., 2007), as well as the existence in plants of plant-specific immunological synapses (Baluška et al., 2005; Kwon et al., 2008; Sup Yun et al., 2008). Memories of gravity- and photo-stimulations (Nick et al., 1990; Nick and Schafer, 1998) should also be included in future studies.

## Conclusions and outlook

Plants live in complex environments and their survival is dependent on the reliable sampling of critical biotic and abiotic parameters. To this end, plants and their roots use their sensory systems to sample and integrate the complex sensory information from their environment for the sake of responding adaptively. Similarly, as in higher animals and humans (Laughlin, 2014; Picard and Friston, 2014), faithful representation of their outside world into their metabolic, physiological and behavioral adaptations are essential for their survival. To achieve this goal via optimization of their Darwinian fitness, plants use their own plant-specific intelligence, cognition, and behavior (Trewavas, 2005, 2014; Karban, 2008; Baluška and Mancuso, 2009a,b; Baluška et al., 2009; Karpiński and Szechyńska-Hebda, 2010; Calvo Garzón and Keijzer, 2011; Trewavas and Baluška, 2011; Marder, 2012, 2013; Gagliano et al., 2014; Cvrčková et al., 2016).

Although it might be tempting, on first glance, to relate plant cognition (Trewavas, 2002) to fitness, or as the slogan goes, to the “the survival of the fittest,” we need something other than natural selection *per se* to grasp plant intelligence. Evolving an adaptation and, say, learning, are different things, but the inheritance-variation-selection view of the evolution of intelligence leaves out of the picture the very learning that takes place in ontogeny. Different individual plants, despite sharing their genotype (those that belong to the same species), can still develop markedly different phenotypes as a function of diverging environmental demands and organismal epigenetic control. Signal integration encompasses phylogenetic endowment coupled with ontogenetic exposures. Individual plants do learn particularities of their local environment (Silvertown and Gordon, 1989; Gagliano et al., 2014; Gagliano, 2015) that could not possibly be predicted on an evolutionary timescale.

The predictive coding model hypothesizes that cascades of sensory input can be surprising to both animals and plants, generating free energy (prediction error). As a result, plants like animals may perform active inference. Recall the example of the fresh-water fish that might minimize free energy by re-sampling the environment in order to bring future input into line with expectations. Our hypothesis is that this may well be the case with salt-avoidance behavior in plants. Integrated environmental assessment (salinity, gravitropic, etc.) appears to be needed at the root level in order to optimize growth under abnormal saline conditions. This means that the plant root, like the fish, would follow the salt-water gradient back into fresh water. If roots enjoy salt-avoidance behavior then this implies an intelligent adaptive strategy that calls for the active, salt-induced, modification of root growth (Li and Zhang, 2008; Sun et al., 2008; Yokawa et al., 2014). Future studies will illuminate how the plant-specific sensory systems feed first into the bioelectrical phenomena at the plasma membrane, and into cellular processes underlying adaptive responses, and behavior at the subcellular, cellular and supracellular organ levels. Plant roots will serve well in these studies as the root apex is an exploratory plant organ, endowed with the sensory root cap, optimized for the search of plant nutrients and the avoidance of toxic and dangerous soil patches.

Once anthropocentric preconceptions are superseded, we may label plant behavior as cognitive insofar as it is flexible and adaptive. With that being said, it goes without saying that predictive coding is not the one and only alternative to feature detection. Unlike predictive coding-based strategies, an ecological, non-model-based approach (Calvo et al., 2014) may exploit information available at the level of the coupling itself between organism and environment, telling against top-down flows of representational predictions that operate in predictive-coding. For the sake of direct comparison, we have only considered “feature detection” vs. “predictive coding” models of plant behavior. Whether a continuous coupling of perception-action that may account for forms of anticipation in non-representational terms is plausible remains an open question for future research^6^.

## Author contributions

All authors listed, have made substantial, direct and intellectual contribution to the work, and approved it for publication.

### Conflict of interest statement

The authors declare that the research was conducted in the absence of any commercial or financial relationships that could be construed as a potential conflict of interest.

## Footnotes

^1^Thanks to an anonymous referee for *Frontiers* for pressing on the lack of an *experimentum crucis*, and to Tony Chemero for bringing this third possibility to our attention. See our point about model-free vs. model-based modes of prediction in Section Expectation and Surprise in the Ventral Visual Pathway, and some more specific empirical suggestions in Section Applying the Experimental Paradigm to Plants.

^2^The fact that events being coded for by detectors are hard-wired does not entail that no learning can possibly take place (see end of this section).

^3^Note that Egner et al. (2010) “assume FFA prediction error units to be activated by positive prediction error (the occurrence of an unexpected face), but not by negative prediction error (the unexpected omission of a face, corresponding to an unexpected house stimulus” Egner et al., 2010, p. 16604).

^4^There is an objection against the idea of active inference which states that the idea entails that a surprise minimizing organism can best do this by retreating from all sensory stimuli; that is, by cutting themselves off from the world inside a “dark room.” The appropriate reply to this objection is that the dark room is only unsurprising under the assumption that the organism has no expectations about its future states (cf., Friston et al., 2012b).

^5^We thank an anonymous reviewer for bringing this point to our consideration.

^6^For preliminary results see Calvo et al. (2015).
